# Spore Acquisition and Survival of Ambrosia Beetles Associated with the Laurel Wilt Pathogen in Avocados after Exposure to Entomopathogenic Fungi

**DOI:** 10.3390/insects9020049

**Published:** 2018-04-25

**Authors:** Pasco B. Avery, Verónica Bojorque, Cecilia Gámez, Rita E. Duncan, Daniel Carrillo, Ronald D. Cave

**Affiliations:** 1 Indian River Research and Education Center, IFAS, University of Florida, 2199 South Rock Road, Ft. Pierce, FL 34945, USA; veroboj@hotmail.com (V.B.); cgam.h@hotmail.com (C.G.); rdcave@ufl.edu (R.D.C.); 2 Escuela Agrícola Panamericana, P.O. Box 93 Tegucigalpa, Honduras; 3 Tropical Research and Education Center, IFAS, University of Florida, Homestead, FL 33031, USA; ritad@ufl.edu (R.E.D.); dancar@ufl.edu (D.C.)

**Keywords:** ambrosia beetles, laurel wilt, avocados, entomopathogenic fungi, Kaplan–Meier analysis

## Abstract

Laurel wilt is a disease threatening the avocado industry in Florida. The causative agent of the disease is a fungus vectored by ambrosia beetles that bore into the trees. Until recently, management strategies for the vectors of the laurel wilt fungus relied solely on chemical control and sanitation practices. Beneficial entomopathogenic fungi (EPF) are the most common and prevalent natural enemies of pathogen vectors. Laboratory experiments demonstrated that commercial strains of EPF can increase the mortality of the primary vector, *Xyleborus glabratus*, and potential alternative vectors, *Xylosandrus crassiusculus*, *Xyleborus volvulus* and *Xyleborus bispinatus* (Coleoptera: Curculionidae: Scolytinae). Our study provides baseline data for three formulated commercially-available entomopathogenic fungi used as potential biocontrol agents against *X. crassiusculus*, *X. volvulus* and *X. bispinatus.* The specific objectives were to determine: (1) the mean number of viable spores acquired per beetle species adult after being exposed to formulated fungal products containing different strains of EPF (*Isaria fumosorosea*, *Metarhizium brunneum* and *Beauveria bassiana*); and (2) the median and mean survival times using paper disk bioassays. Prior to being used in experiments, all fungal suspensions were adjusted to 2.4 × 10^6^ viable spores/mL. The number of spores acquired by *X. crassiusculus* was significantly higher after exposure to *B. bassiana*, compared to the other fungal treatments. For *X. volvulus*, the numbers of spores acquired per beetle were significantly different amongst the different fungal treatments, and the sequence of spore acquisition rates on *X. volvulus* from highest to lowest was *I. fumosorosea* > *M. brunneum* > *B. bassiana*. After *X. bispinatus* beetles were exposed to the different suspensions, the rates of acquisition of spores per beetle amongst the different fungal treatments were similar. Survival estimates (data pooled across two tests) indicated an impact for each entomopathogenic fungus per beetle species after exposure to a filter paper disk treated at the same fungal suspension concentration. Kaplan–Meier analysis (censored at day 7) revealed that each beetle species survived significantly shorter in bioassays containing disks treated with EPF compared to water only. This study demonstrated that ambrosia beetles associated with the laurel wilt pathogen in avocados are susceptible to infection by EPF under laboratory conditions. However, the EPF needs to be tested under field conditions to confirm their efficacy against the beetles.

## 1. Introduction

Ambrosia beetles have symbiotic relationships with fungi that contribute to their ecological, biological and economic importance [1,2,3]. Ambrosia beetles associated with plant pathogenic fungi that cause diseases, such as *Raffaelea lauricola* T.C. Harr., Fraedrich & Aghayeva and *Fusarium* spp., are becoming relevant pests for the forestry and agricultural sector in many countries [4,5,6]. For example, ambrosia beetles vector the fungus *R. lauricola*, which can cause laurel wilt disease in redbay, *Persea borbonia* (L.), silk bay, *P. humilis* Nash, swamp bay, *P. palustris* (Raf.) Spreng., and avocado, *Persea americana* Mill. [7,8,9]. Trees infected with *R. lauricola* are characterized by vascular discoloration, rapid wilting, defoliation, necrosis of foliage and sometimes xylem dysfunction [10]. The ability of some ambrosia beetles to act as vectors of plant pathogenic fungi in trees has brought much attention for finding management strategies that could reduce the impact of these insects.

Until recently, management strategies for ambrosia beetles in avocados have relied primarily and intensively on spraying broad-spectrum chemical insecticides, but with limited success, especially when targeting beetles that were already inside the tree [11]. Biological control using entomopathogenic fungi has been successful for managing bark beetle pests of economic importance worldwide and could also play a relevant role in the management of ambrosia beetles in avocados, as well [12,13,14,15,16]. Carrillo et al. [15] found that the fungal product BotaniGard^®^ ES containing the entomopathogenic fungus *Beauveria bassiana* (Balsamo) had the highest efficacy and percentage mycosis compared to other fungal products tested against the red bay ambrosia beetle, *Xyleborus glabratus* Eichhoff, under laboratory conditions. At the time this research was conducted, it was assumed that *X. glabratus* was the only vector carrying *R. lauricola* to the avocados [17]; however, after further research and observations in the field, Carrillo et al. [18] found that *X. glabratus* is rare in commercial avocado crops. By contrast, several species of ambrosia beetles are associated with *R. lauricola* in avocado production systems. *Xyleborus volvulus* (Fabricius, 1775) and *Xyleborus bispinatus* Eichhoff (formerly recognized as *X. ferrugineus* (Fabricius) [19]) were demonstrated capable of transmitting the pathogen to healthy avocado trees [8,9]. The ambrosia beetle *Xylosandrus crassiusculus* (Motschulsky) has also been found carrying the pathogen in its mycangial pouches and attacking avocado trees [9]. Therefore, this species is still considered a potential vector of the laurel wilt disease pathogen, although it has not yet been confirmed.

The objective of our study was to create baseline data for three commercially-formulated entomopathogenic fungi that may be used as potential biocontrol agents against *X. crassiusculus*, *X. volvulus* and *X. bispinatus.* The specific objectives were to parameterize: (1) the number of viable spores acquired per beetle species and (2) the median and mean survival times of *X. crassiusculus*, *X. volvulus* and *X. bispinatus* adults after exposure to formulated fungal products containing different strains of the entomopathogenic fungi *Isaria fumosorosea* (Wize), *Metarhizium brunneum* (Petch) and *B. bassiana* in paper disk bioassays.

## 2. Materials and Methods

### 2.1. Beetles

Adults of *X. bispinatus* were obtained from a laboratory colony maintained following the procedures described by Menocal et al. [20]. Adults of *X. crassiusculus* and *X. volvulus* were obtained from infested avocado trees as described by Carrillo et al. [15]. Collected female beetles that emerged from the wood were collected daily and placed inside the Petri dishes (5 cm diameter) provided with a moistened filter paper, and the lid was closed, forming a chamber. Closed dish chambers containing the beetles were then sealed with Parafilm^®^ (Bemis Co., Inc., Neenah, WI, USA) and transported overnight to the University of Florida, Indian River Research and Education Center at Fort Pierce, Florida. Upon arrival, dishes were immediately unsealed, lids briefly opened (~30 s) and closed and then placed on the laboratory bench for ~2–3 h. This procedure allowed the beetle’s time to acclimate to room temperature before being used in the bioassays. All adult females in the bioassays were used within 2 days of collection.

### 2.2. Fungi

One commercial strain of *M. brunneum* (Met52^®^ EC) isolated from the codling moth, *Cydia pomonella* (L.) (J. Leland, personal communication, Novozymes Biologicals, Inc., Salem, VA, USA), one commercial strain of *I. fumosorosea* (PFR-97^®^ 20% WDG) isolated from the mealybug *Phenacoccus solani* Ferris in Apopka, FL (Certis USA, Columbia, MD, USA) and one commercial strain of *B. bassiana* GHA (BotaniGard^®^ ES, Laverlam International, Butte, MT, USA) were tested. All products were refrigerated at 4 °C until needed for experiments.

### 2.3. Determining Fungal Suspension Spore Density and Viability

The PFR-97 suspension was prepared by mixing 1 g of the blastospore-containing powder in 100 mL of sterile distilled water, stirring with a magnetic bar for 30 min and then letting the inert material precipitate for an additional 30 min, leaving the supernatant containing the blastospores. Both BotaniGard ES and Met52 EC conidial suspensions were each prepared by mixing 1 mL of the oil-emersion liquid product in 100 mL of distilled water and stirring the conidial suspension with a magnetic bar for 15 min. All conidial or blastospore suspension densities were measured by using a plastic disposable C-Chip Neubauer Improved hemocytometer (Incyto DHC-NO 1, Chungnam, Korea) observed under a Leica DM500 Brightfield microscope (Leica Microsystems, Wetzlar, Germany) at 400× magnification. All fungal suspensions were adjusted to ~2.4 × 10^6^ blastospores or conidia/mL after being diluted with distilled water using the following formula: Fc x Fv = Sc x Sv, where Fc = final concentration, Fv = final volume, Sc = stock concentration and Sv = stock volume. Changing focal depth with the microscope was required to distinguish *B. bassiana* and *M. brunneum* conidia, which have conspicuous cell walls, from droplets formed after adding water to the emulsified suspension in the BotaniGard ES and Met52 EC formulation, respectively.

Viability was determined using percent germination for all strains by spreading 100 μL of each suspension containing 10^6^ blastospores or conidia/mL on Petri dishes containing potato dextrose agar (PDA) with streptomycin (0.5%) added. Dishes were sealed with Parafilm^®^, transferred to the growth chamber and incubated at 25 °C for 18–24 h under a 14-h photophase. Percentage germination per fungal strain was determined by observing 100 spores in two different areas (200 spores/plate) using a Leica DM500 Brightfield microscope (400×) on triplicate PDA plates. Germination was indicated once a germ tube had formed and was at least half the length of the blastospore or conidia. The number of viable spores/mL was determined using the following formula:

Number of viable spores/mL = total number of spores/mL × % germination, where % germination = number of spores germinated/200 total spores counted per plate × 100.(1)

### 2.4. Determining the Acquisition of Spores by Each Beetle Species per Fungal Suspension

Ten adult beetles of each species were tested per fungal strain per treatment. Beetles were placed individually in a single well of a sterile polystyrene 12-well Costar^®^ cell culture cluster plate (Corning Inc., Corning, NY, USA), containing 0.5 mL of either blastospore or conidial fungal suspensions (~2.4 × 10^6^ viable spores/mL), for 1–2 min to become contaminated. Contaminated beetles were then placed individually in Eppendorf tubes (1.5 mL) containing 200 μL of Triton X-100 and vortexed for 15 s. After being vortexed, one aliquot (10 μL) of each suspension was placed in the C-Chip hemocytometer, and the mean number of viable spores/mL per beetle was determined as described above. Beetles placed in water only, vortexed and then spores counted as above served as a control. Five beetles were used per treatment, and the experiment was conducted twice.

### 2.5. Filter Paper Disk Bioassays Chambers and Survival Assessment

Bioassay chambers used in this experiment consisted of polystyrene plastic vials (88 oz: 1 1/16″ Ø × 2 3/16″H, Tap Plastics^®^, San Leandro, CA, USA) that contained a moistened filter paper disk (15 mm Ø). A 1-cm Ø hole was cut in the snap cap and a piece of cotton rope inserted, both for ventilation, as well as for maintaining additional moisture inside the vial.

**Figure 1:**
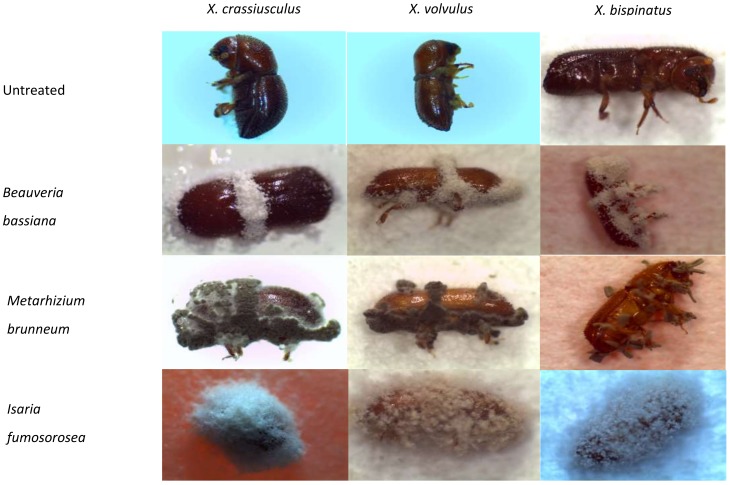
Comparison of untreated and mycosed *Xylosandrus crassiusculus*, *Xyleborus volvulus* and *Xyleborus bispinatus* after exposure to a filter paper disk inoculated with fungal suspensions (2.4 × 10^6^ spores/mL) of either *Beauveria bassiana* (BotaniGard ES), *Metarhizium brunneum* (Met52 EC), or *Isaria fumosorosea* (PFR-97 20% WDG). Untreated = control disks with distilled water only. Fungal phenotype post-mortem for mycosed beetles: *Beauveria bassiana* with white spores; *Metarhizium brunneum* with green spores; *Isaria fumosorosea* with white to greyish mauve spores.

The filter paper disk inside each vial prior to exposure to the beetle was contaminated with 100 μL of one of the fungal suspensions. Disks moistened with only distilled water were used as controls. After the disks were treated, 15–25 untreated beetles of each species (number based on the availability of beetles/treatment/experiment) were immediately placed individually in the vial bioassay chambers, and the snap cap with the moistened cotton rope was replaced. All vials in a randomized block design were held upright in high density polystyrene racks incubated at 25 °C under a 14-h photoperiod and examined every 24 h until all beetles died. Each cotton rope was moistened to saturation and 30 μL of distilled water were added to the filter paper disk daily. Dead beetles were removed daily, surface sterilized using the procedure described by Lacey and Brooks [21] and transferred to Petri dishes containing moistened filter paper or 1.4% water agar. Dishes were sealed with Parafilm^®^ and incubated the same as above. Beetles were examined 7–10 days post-mortem for mycosis, and the fungal phenotype was verified (Figure 1). Experiments were conducted twice.

### 2.6. Statistical Analysis

The mean number of spores acquired and days to death for each beetle species after exposure to each fungal treatment compared to the control were statistically analyzed for significance using a one-way ANOVA (*p* < 0.05). If significant, treatment post hoc means were separated using Tukey’s HSD test (*p* < 0.05). Statistical analyses were conducted using SAS Proc GLM procedures and executed on a PRO_WIN 6.1 platform (SAS 2002–2012; SAS Institute Inc., Cary, NC, USA). Median survival times (ST_50_) were compared through Kaplan–Meier survival analysis followed by a log rank test (SAS JMP 8 for Windows 2013).

## 3. Results

### 3.1. Fungal Suspension Spore Density and Viability

Spore densities and percentages of germination adjusted to produce 2.4 × 10^6^ viable spores/mL for each fungal suspension are presented in Table 1. Spore densities were very similar, and the germination rate was only slightly higher for *B. bassiana*; however, all fungal suspensions were adjusted to 2.4 × 10^6^ viable spores/mL prior to use in the experiments.

### 3.2. Acquisition of Spores by Each Beetle Species per Fungal Suspension

The number of spores acquired after being dipped into a fungal suspension varied greatly among beetle species (Table 2). Spore acquisition by *X. crassiusculus* was significantly higher after exposure to *B. bassiana*, compared to the other fungal treatments. For *X. volvulus*, spore acquisition was highest with *I. fumosorosea* and lowest with *B. bassiana*. After *X. bispinatus* beetles were exposed to the different suspensions, spore acquisition per beetle did not differ significantly among the fungal treatments. None of the beetles in the control were contaminated with any of the fungal species tested.

### 3.3. Survival after Exposure to Each Fungal Treatment in a Filter Paper Disk Bioassay

**Figure 2:**
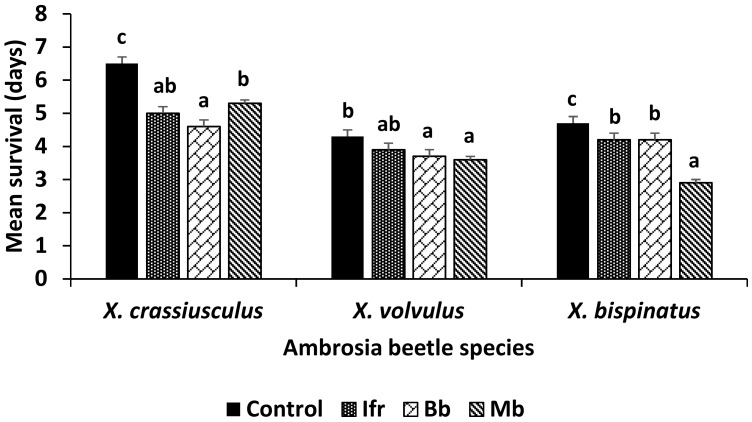
Comparison of the mean (± SEM) days of survival for *Xylosandrus crassiusculus* (*n* = 30 per treatment), *Xyleborus volvulus (n* = 50 per treatment) and *Xyleborus bispinatus* (*n* = 40 per treatment) after exposure to a filter paper disk treated with 100 μL of one of three fungal suspensions (2.4 × 10^6^ spores/mL) or distilled water for seven days. Control = distilled water only, Ifr = *Isaria fumosorosea* (PFR-97 20% WDG), Bb = *Beauveria bassiana* (BotaniGard ES), Mb = *Metarhizium brunneum* (Met52 EC). Bars for each beetle species not followed by the same letter are significantly different (Tukey’s HSD test, *p* < 0.05).

Survival rates (data pooled across both tests) indicated an impact by each entomopathogenic fungus on all beetle species after exposure to a filter paper disk treated at the same suspension concentration (Figure 2). Kaplan–Meier analysis (censored at day 7) revealed that each beetle species survived significantly longer in bioassays with disks inoculated with water only compared to entomopathogenic fungi, i.e., *X. crassiusculus*: log rank *X*^2^ = 45.0, *p* < 0.0001, df = 3; *X. volvulus*: log rank *X*^2^ = 13.2, *p* = 0.0043, df = 3; *X. bispinatus*: log rank *X*^2^ = 42.7, *p* < 0.0001, df = 3. The ST_50_ (days) for water only, *I. fumosorosea*, *B. bassiana* and *M. brunneum* treatments, respectively, after each beetle species was exposed to a paper disk for seven days, were as follows: *X. crassiusculus*: 5.6, 4.9, 4.5, 5.3; *X. volvulus*: 4.3, 3.9, 3.7, 3.6; *X. bispinatus*: 4.7, 4.3, 4.2, 2.9. The mean survival time for *X. crassiusculus* beetles after exposure to each fungal suspension treatment was significantly (*F* = 34.9; df = 3, 87; *p* < 0.0001) shorter compared to the control (water only) (Figure 2). *Xylosandrus crassiusculus* beetles exposed to *B. bassiana* died faster than when exposed to *M. brunneum*; however, days survival for *I. fumosorosea* were similar to both *B. bassiana* and *M. brunneum*. For *X. volvulus*, mean survival times were significantly (*F* = 4.36; df = 3, 147; *p* = 0.0057) shorter for beetles exposed to *M. brunneum* and *B. bassiana* compared to water only; survival time of beetles exposed to *I. fumosorosea* were similar to the other treatments and control. The mean survival time of *X. bispinatus* beetles exposed to *M. brunneum* was significantly (*F* = 63.1; df = 3, 117; *p* < 0.0001) shorter than that of beetles exposed to *I. fumosorosea* and *M. brunneum*; mean survival time in the control was significantly longer than in the fungal treatments.

## 4. Discussion

This study demonstrated the efficacy of entomopathogenic fungi (EPF) against adult *X. bispinatus* and *X. volvulus* and assessed their actual dose delivered by immersion. In addition, this study adds further support to the original finding that *X. glabratus* and the three ambrosia beetles tested in this study are susceptible to the same entomopathogenic fungal biopesticides that are commercially available nationwide for avocado growers to use in their pest management programs.

Adult ambrosia beetles seemingly vary considerably in their ability to acquire the different types of spores suspended in the formulated biopesticide products. In our study, the number of acquired spores per *X. bispinatus* adult was similar amongst the three fungal treatments. In contrast, *X. volvulus* and *X. crassiusculus* acquisition did differ significantly among the fungal products. This variation in spore acquisition per beetle may be related to the morphological differences of each species and therefore cannot be compared across species due to their size and setal density differences. This hypothesis was not the focus of our study and cannot be confirmed from the results of this study, because the number of spores per mm^2^ area of the cuticle was not determined, and would require further research. In our study, the differences in spore acquisition may also be related to the type of propagule and the formulation of the fungal product.

Carrillo et al. [15] found that the number of spores acquired by *X. glabratus* after being dipped in the same fungal products also varied significantly. They suggested that the variance in the number of spores acquired per beetle species may also be due to the adult’s physicochemical surface properties of its cuticle, as well as the fungal propagules. The insect cuticle is typically hydrophobic [22], and the adhesion of the fungal propagule, i.e., conidia or blastospore, is determined primarily by hydrophobic interactions on the surface [23]. Both BotaniGard and Met52 contain aerial conidia that are hydrophobic [24,25], and PFR-97 contains blastospores that are hydrophilic [26]. The hydrophobic conidia initially have greater non-specific binding to the hydrophobic cuticle than the hydrophilic blastospores [27]. However, due to the hydrophobic properties of *B. bassiana* and *M. brunneum* conidia compared to the hydrophilic properties of the *I. fumosorosea* blastospores on the beetle surface, it is possible that after washing the spores off of the beetle surface using the surfactant Triton X-100 (0.01% *v*/*v*) that this procedure may not have removed all the blastospores adhering to the beetle cuticle, which may account for variations in the number of spores recovered for PFR-97 treatments compared to BotaniGard and Met52 from some of the beetle species. This hypothesis still warrants more research, especially with reference to the effect of hydrophilic properties and the adherence of *I. fumosorosea* blastospores to the insect cuticle when using this washing technique for removing blastospores from the insect cuticle.

For the control, distilled water was used only, which is a realistic control to assess the efficacy of PFR-97 (*I. fumosorosea*), which is a dry desiccation-tolerant blastospore-formulated powder with no surfactants or emulsifiers added to the formulation. When PFR-97 is mixed in water, the spores will suspend immediately in the supernatant; however, this is not the case for BotaniGard ES (*B. bassiana*) or Met52 EC (*M. brunneum*). Both of these conidial formulated fungal products contain a proprietary mixture of surfactants and emulsifiers suspended in oil that allows the spores to suspend when mixed in water. Therefore, in future bioassays, water only and the resultant supernatant minus the spores would be a more appropriate control when assessing the efficacy of these commercially-formulated fungal products.

Adult beetles in our study using the paper disk bioassay technique were exposed to the formulated fungal products in two ways, either indirectly by residual contact or directly by *per os* inoculation, i.e., ingesting or acquiring the spores while chewing the inoculated disk. All of the adult beetles chewed on the paper disk in the bioassay chamber and had the opportunity to obtain spores *per os*, as well as acquire spores on their body parts as they roamed on the inoculated disk. Fernandez et al. [28] observed that the second instar of the Colorado potato beetle, *Leptinotarsa decemlineata* (Say), acquired a high density of *B. bassiana* conidia on the legs, mouthparts and ventral surface areas when roaming on sprayed potato foliage. Although the Colorado potato beetle larva was contaminated, they suggested that mortality probably occurred because of infection through the cuticle of the beetle and not by ingesting *B. bassiana* conidia while feeding on the leaf surface. Therefore, in our study, the adherence of propagules obtained from the spore residue on the paper disk may have played a significant role in mortality and its efficacy may be directly related to the formulation specifics of each of the fungal products. Although speculative, this hypothesis warrants further research to elucidate the role that each inoculation process may play in the overall mortality of the different adult ambrosia beetle species.

In our study, the mean survival of the different ambrosia beetle species per formulation varied similarly to that of their spore acquisition. Beetles exposed to the disks inoculated with *B. bassiana* and *M. brunneum* died significantly faster than those with *I. fumosorosea*. As stated above, BotaniGard and Met52 are both oil/emulsifier-formulated, and PFR-97 is a blastospore powder-type product. The *B. bassiana* and *M. brunneum* treatments, when applied in oil-in-water emulsion, will have conidia generally surrounded by oil droplets, which may enhance their adherence to the insect cuticle. Prior et al. [29] demonstrated that *B. bassiana* conidia in an oil-based formulation adhered significantly better to the cuticle of the cocoa weevil, *Pantorhytes plutus* (Oberthür), and enhanced higher mortality with a lower lethal time value compared to the water formulation. Under laboratory and field conditions, Batta [30] demonstrated a significantly higher level of efficacy with formulated *B. bassiana* conidia in an emulsion compared to no emulsion against the almond bark beetle, *Scolytus amygdali* Guérin-Méneville. In another study, Carrillo et al. [15], using the same paper disk bioassay technique described above at the same suspension concentration per fungal product, found that the oil/emulsifier-formulated BotaniGard and Met52 had a higher mortality compared to the *I. fumosorosea* blastospore-powder formulated PFR-97 product and water only. Overall, oil-based formulations of fungal conidial biopesticides have been reported to increase the adherence of propagules to the insect integument by: (1) enhancing the spread of the inoculum and subsequent penetration of the insect cuticle, (2) protecting propagules from ultraviolet light radiation and (3) enhancing infection under low humidity [31]. Factors that may inhibit or enhance germination and penetration include cuticle density or compounds on the insect integument [32,33]. In addition, the lack of nutrients on sclerotized beetle cuticle is a limiting factor in fungal growth and development [34]. Other physical or chemical aspects defining the interactions at the cuticle barrier between the entomopathogenic fungus and insect that ultimately can lead to either successful mycosis by the entomopathogen or successful defense by the host have been extensively reviewed [35]. Our data suggest that the formulation of each EPF can affect the adherence of spores to beetles, which could have a subsequent positive effect on reducing the spread of *R. lauricola*. Considering that these bioassays were conducted under optimum laboratory conditions, the apparent positive effect of the conidial oil-based formulations remains to be confirmed under field conditions.

The ambrosia beetle species that are vectors of *R. lauricola* are morphologically different, but their reproductive behavior should be similar to that of *X. glabratus*. Ideally, EPF treatments could prevent ambrosia beetles from boring into the trees, thereby potentially eliminating the risk of *R. lauricola* transmission. However, Carrillo et al. [15] found that that *X. glabratus* females were able to bore into avocado log bolts and construct galleries regardless of the fungal treatment. Carrillo et al. [15] also observed that even if the EPF-infected beetle bores into the tree, the mycosed insect inside the tree will kill the next-generation brood. Brar et al. [36] observed that *X. glabratus* females excavate a primary gallery that can branch into secondary and tertiary galleries where eggs are laid, ~7 days after gallery initiation. The median and mean survival times of *X. glabratus* and the other ambrosia beetle females in our study exposed to EPF ranged from 3–5 days based on a dose of 2.4 × 10^6^ spores/mL. This spore concentration tested under laboratory conditions is much lower compared to a typical rate applied in the field (e.g., 8.8 × 10^7^ spores/mL for BotaniGard ES), yet it provided efficacious mortality. Although all tested fungal formulated products can prevent beetle reproduction, the most effective fungal product will be the one causing the highest beetle mortality regardless of their survivorship times. In addition, the entomopathogenic fungal infection of adult ambrosia beetle females could potentially suppress the growth or establishment of their fungal symbionts in the galleries that become contaminated with entomopathogenic and saprophytic organisms associated with the beetle cadavers. Results from laboratory competition *in vitro* studies indicate that the biofungicide product RootShield^®^ Plus^+^WP, containing *Trichoderma harzianum* Rifai and *T. virens* (J.H. Mill., Giddens & A.A. Foster), can inhibit the radial hyphal growth of *R. lauricola* (P. Avery, unpublished data). Perhaps, this biofungicidal product could be applied on the trunk of the avocado trees along with the EPF and potentially reduce the spread of *R. lauricola* in the groves. This promising strategy warrants much more research for determining the most efficacious application of the selected biopesticides under laboratory and then field conditions.

In our experiments, beetles were offered the inoculated paper disk in a no-choice condition and exposed to EPF over time. This same paper disk bioassay technique was used by Carrillo et al. [15] for testing the same EPF against *X. glabratus* which resulted in similar findings to our study. Although our findings using this technique showed significant differences among treatments and the control, the short mean longevity of beetles in the controls (~7–8 days) and relatedness to the natural conditions in the field were a concern when assessing the efficacy of these fungal products. The poor survival of *X. volvulus* and *X. bispinatus* beetles could be attributed to the fact that the inoculated paper disk in each vial did not remain as moist throughout the day as those with the *X. crassiusculus* beetles. During the bioassay trials with *X. crassiusculus* beetles, the inoculated disks were watered twice a day, whereas the disks that the *X. volvulus* and *X. bispinatus* beetles were exposed to were watered once due to the lack of labor availability. Therefore, due to the concern noted above, an improved avocado bark plug bioassay is being designed for exposing the beetles to each fungal product under semi-natural field conditions (P. Avery, personal communication).

Due to the similarity of the life histories and behavior of *X. crassiusculus*, *X. volvulus* and *X. bispinatus* to *X. glabratus*, we will use *X. glabratus* as a model system because it has been studied extensively compared to the other beetles [36]. The model system will be used to visually describe a possible scenario where using EPF as a pest management strategy can be more efficacious than just applying conventional insecticides. According to observations by Brar et al. [36], these ambrosia beetles have a unimodal dispersal peak, engaging in host-seeking flight during the late afternoon, which is directed by plant host-based volatiles, primarily sesquiterpenes [37]. Upon finding a host tree, the beetles roam on the bark of the tree trunk for relatively long periods of time (~1 h, D. Carrillo, personal observation) before boring into the trunk. During the roaming time, the beetles might be exposed to fungal spores by landing on an EPF-contaminated bait station or bark surface of a host tree prior to engaging in another host-seeking flight. Carrillo et al. [15] found high infection rates in beetles interacting with wood treated with the different fungi and suggested that trunk sprays or bait stations where the beetles land and interact with a fungus-treated surface could act as delivery and auto-dissemination systems. Improving the delivery system of fungal strains could result in increased mortality rates of ambrosia beetle females.

*Xyleborus volvulus* and *X. bispinatus* are confirmed vectors of *R. lauricola* that can kill an avocado tree by the inoculation of a few spores. This transmission of the pathogen definitely increases the challenge of achieving an integrated pest management approach and thereby the probability that conventional insecticides will need to be applied often by avocado growers. The current pest management strategy is based on early detection and removal of diseased trees to eliminate beetle breeding sites and fungal inoculum sources. The diseased trees are uprooted, stump and roots burned, the trunk and limbs chipped and the chips and adjacent trees sprayed with synthetic insecticides. Our results suggest that applications of EPF, in combination with other management tactics, including the use of compatible conventional pesticides, might suppress beetle populations that vector *R. lauricola* to avocados and mitigate the adverse effects of this beetle-disease complex. In another study, work is ongoing to determine the conventional agrochemicals commonly used by growers that are compatible with the commercially-available fungal biopesticides containing EPF, which can be applied together for management of the ambrosia beetles vectoring *R. lauricola* to avocado trees.

## 5. Conclusions

In field experiments, Carrillo et al. [15] revealed that EPF do not prevent *X. glabratus* beetles from boring into the trees; however, the EPF-infected beetles die inside the trees and mycose without reproducing. Therefore, the EPF may provide control similar to that by chemical insecticides, but persist longer on the bark of avocado trees and will potentially cause epizootics under the proper environmental conditions. Our study demonstrated that other ambrosia beetle vectors of *R. lauricola* that have been observed on the avocados are also susceptible to infection by EPF and should be able to be managed effectively. Therefore, to improve field efficacy, EPF must be integrated into the management strategy for *R. lauricola* and other avocado pests, which includes the use of several fungicides, insecticides and adjuvants. In another study, we are determining antagonistic and synergistic relationships among EPF and agrochemicals used by avocado growers. Several producers have incorporated this biocontrol strategy to manage ambrosia beetles and other pests of avocado, but the main limitation of EPF is they do not control ambrosia beetles already breeding inside the trees. Therefore, the search for improved delivery systems that could increase the efficacy of EPF, as well as the integration of biofungicides for reducing the spread of laurel wilt disease is still ongoing.

## Figures and Tables

**Table 1:** Spore densities and viabilities of three entomopathogenic fungal suspensions.

Treatment ^a^	Spores/mL (×10^6^)	Germination (%)	Viable Spores/mL (×10^6^)
*I. fumosorosea*	2.9 ± 0.42	85	2.4 ± 0.36
*M. brunneum*	2.8 ± 0.27	85	2.4 ± 0.23
*B. bassiana*	2.7 ± 0.37	89	2.4 ± 0.33

^a^*Isaria fumosorosea* (PFR-97 20% WDG), *Metarhizium brunneum* (Met52 EC) and *Beauveria bassiana* (BotaniGard ES).

**Table 2:** Comparison of the acquisition of spores by three ambrosia beetle species after being dipped into three entomopathogenic fungal suspensions ^a^.

	No. of Spores (x 10^5^)/beetle ^c,d^		
Treatment ^b^	*X. crassiusculus*	*X. volvulus*	*X. bispinatus*
*I. fumosorosea*	4.5 ± 0.4 ^a^	4.5 ± 0.4 ^c^	0.9 ± 1.0 ^a^
*M. brunneum*	4.0 ± 0.4 ^a^	2.9 ± 0.3 ^b^	3.8 ± 1.2 ^a^
*B. bassiana*	8.2 ± 0.4 ^b^	1.1 ± 0.2 ^a^	2.4 ± 0.4 ^a^
Statistical Analysis	*F* = 11.3; df = 2.8; *p* = 0.0018	*F* = 26.2; df = 2.8; *p* < 0.0001	*F* = 3.35; df = 2.8; *p* = 0.0878

^a^ All fungal suspensions were adjusted to 2.4 × 10^6^ spores/mL. ^b^ *Isaria fumosorosea* (PFR-97 20% WDG), *Metarhizium brunneum* (Met52 EC), *Beauveria bassiana* (BotaniGard ES), control = distilled water only. ^c^ Individual beetles were placed in an Eppendorf tube with 200 µL of 0.1% Triton X-100 after being exposed to the specific fungal suspension or water and vortexed for 15 s. ^d^ Values not followed by the same letter in a column are significantly different (Tukey’s HSD test, *p* < 0.05).
